# Influences of Flipped Teaching in Electronics Courses on Students’ Learning Effectiveness and Strategies

**DOI:** 10.3390/ijerph18189748

**Published:** 2021-09-16

**Authors:** Chih-Cheng Lo, Ming-Hsien Hsieh, Hsiao-Hsien Lin, Hsu-Hung Hung

**Affiliations:** 1 Department of Industrial Education and Technology, National Changhua University of Education, Changhua County 500, Taiwan; d0831013@mail.ncue.edu.tw; 2 School of Physical Education, Jiaying University, Meizhou 514015, China; 3 National Erh-lin Industrial and Commercial Vocational High School, Division of Continuing Education, Erlin Township, Changhua County 500, Taiwan; warrior@kiss99.com

**Keywords:** flipped classroom, learning effectiveness, learning strategies

## Abstract

The potential influence of the COVID-19 pandemic has changed the status of school education and further accelerated the revolution of regular teaching method. This study compared the learning effectiveness and learning strategies of vocational high school students in flipped teaching and traditional teaching modes. By adopting flipped teaching on an electronics course throughout the entire academic year, this study aimed to explore the effect of learning strategies of the students under flipped teaching. The subjects of this study were 85 sophomore students majoring in Electrical Engineering. This study randomly selected one class as the control group (*n* = 43), and adopted the regular teaching method while another class was selected as the experimental group (*n* = 42), and employed the flipped teaching method. This study used the “Learning strategy scale of students in vocational high schools” as the instrument. The students’ scores of the Testing Center for Technological and Vocational Education Test were used to evaluate their learning effectiveness. The results of this study indicate that students under the flipped teaching model made remarkable progress in the electronics course and the learning outcomes remained significant after a long period of time. Moreover, they made notable changes in their learning strategies, including “learning motivation”, “reading and exams”, “self-testing”, and “problem solving strategies”.

## 1. Introduction

The potential influence of the COVID-19 pandemic has changed the status of school education and further accelerated the revolution of regular teaching method. Flipped teaching can be traced back to ancient times when Socrates emphasized that only through interactive communication and debate could students truly acquire knowledge. The teaching approach at that time encouraged students to preview the learning materials before class, and then analyze, summarize, and solve problems during class. Such an approach, which is similar to flipped teaching at present, could facilitate discussions between students and teachers [1].

In the traditional teaching approach, teachers give lessons and students complete exercises, discussions, and assignments after class. The flipped teaching is a different model that asks the students to watch course videos and read textbooks before class, and conduct discussions, exercises, and homework during class [2]. In addition, student-oriented assessment could better evaluate their learning effectiveness. Flipped teaching aims to help students realize their learning situation, and is the basis for students to make progress and a tool for teachers to improve their teaching methods [3]. Flipped teaching places great emphasis on students, and learning motivations play an important role in students’ learning effectiveness. Therefore, this emerging teaching model has attracted the attention of many educators. In his book, 10 reasons to FLIP, there are ten inherent strengths in flipped teaching [4]. First, students could skip or repeat teaching videos based on their own needs. Second, when students do their assignments in class, the teachers could easily identify the students’ learning difficulties, and modify the course contents accordingly to better meet students’ needs. Third, students could obtain various instructions by watching different teaching videos, while teachers could enhance their teaching competency by watching others’ videos. Teachers could thus manage course time efficiently, and parents could engage in the learning process of students and discuss with them after watching the videos. Moreover, various learning theories have evidenced that flipped teaching could improve students’ achievements, interests, and engagement in class, promote the flexible use of technology, and meet the learning demands of the 21st century.

However, electronics is a discipline that highly requires active thinking ability, and students who fail to learn it well would have problems with the development of technical skills in the future. Therefore, as one primary purpose of this study, the researchers intended to explore the influence of teaching strategies on students’ learning effectiveness in the subject of electronics.

In many vocational schools in Taiwan, the flipped teaching model has been applied in arts courses, but seldom to the course of technical subjects. In addition, among related studies, there are scanty studies with 30-week experimental period, even fewer in electronics courses. Therefore, the second purpose of this study is to conduct an in-depth investigation of the effects of flipped teaching in electronics courses. This study mainly explores the following questions. Firstly, have students made remarkable progress in electronics courses under flipped teaching? Secondly, have students shown significant and long-term improvement in the learning of electronics under flipped teaching? Finally, have students made noticeable changes in their learning strategies after participating in flipped teaching?

## 2. Literature Review

This study aims to provide a pedagogical application of flipped teaching models in electronics courses in vocational high school. Previous studies systematically review the influences and positive value of flipped classrooms, and the majority of research is in China, followed by Turkey, Taiwan, and Malaysia [5,6,7,8,9]. For example, Ozudogru and Aksu [8] found that flipped learning affects pre-service teachers’ achievement more than that of the traditional instruction group in Turkey. In addition, Ma et al. [7] show that current studies begin to investigate which features of flipped teaching can significantly improve academic performance, focusing on the changes in students’ learning effectiveness.

Learning effectiveness refers to the changes caused by training and education, including three dimensions, “cognition”, “emotion”, and “action skills” [10], and can be divided into direct and indirect learning effectiveness. Direct learning effectiveness is the change of students’ behavior after instruction, while indirect effectiveness refers to the result reached over a long period of time after instruction. Although many empirical studies might consider that flipped teaching is generally adopted in science, mathematics, or language courses, the innovative applications of flipped teaching, such as nursing education and dance skills [11]. In this paper, flipped teaching can also be used to improve students’ performance in Electronic courses in the vocational high school. The studies included in this Special Issue provide a good demonstration in this regard. Then, the paper constructs a teaching framework following the pattern of flipped teaching model, by highlighting a pedagogical application in long term students’ learning strategies. This framework can provide references for similar courses in vocational high school.

A learning strategy represents the base actions used to acquire, retain, and extract knowledge in the learning process [12], while later learning strategies coordinate and regulate cognition, metacognition, and motivation. Therefore, learning strategies are also called “Self-Regulation Learning” (SRL). Learning effectiveness is the action and process that regulate and control cognition, emotion, behavior, and environment to attain study goals set during the process of acquiring knowledge and skills [13,14]. Moreover, a learning strategy is a kind of learning method and procedure. Learning strategies have been defined as the methods and processes that learners employ to strengthen understanding and improve their learning behaviors [15]. Learning strategies that involve the learners’ practice, motivations, and instrumental perceptions are the actions and processes for knowledge and skills acquisition [16].

The above definitions suggest that learning strategies attach great importance to students’ learning motivations and their own ability to analyze materials, search for clues, and reflect on what they have learned. Passively absorbing knowledge from others is not recommended in the process of learning [17]. The previous study of Hwang et al. shows that dynamic instructional strategies in reading should stimulate critical and creative thinking [18].Therefore, learning strategies in this study highlight the concept of self-regulation leaning and the actions of learning.

In addition to the teaching process, learning strategies also affect the learning outcomes [19]. Learning strategies could improve students’ academic achievements and developments [20]. Over recent years, learning environments have undergone rapid changes, and flipped teaching has become more prevalent. Flipped teaching differs from traditional teaching in the aspect that students are the main characters of the class, while the teachers are assistants. Therefore, the appropriate application of learning strategies is important. When students could review the learning materials repeatedly, organize the learning contents, think independently, and employ cognition strategies to set learning plans, regulate learning methods, make use of resources to manage their time, recognize their learning environment, and ask teachers and peers for help when faced with learning difficulties and problems, the could learn more effectively [13,18].

A number of studies have suggested that, besides the intelligence factor, learning strategies play an important role in distinguishing the learning outcomes of different students. Although students with poor learning effectiveness tend to adopt fewer learning strategies, improving their learning strategies could promote better learning outcomes [20,21,22]. Tang et al. [23] show that the combined model of online teaching with the flipped learning improved students’ learning, attention, and evaluation of courses. Thus, this study aims to discuss whether flipped teaching could improve the learning effectiveness and learning strategies of the students, and even sustain and strengthen their learning strategies.

## 3. Methods

This study applied flipped classroom teaching for electronics courses in a vocational high school, and experimentally explored the influences of flipped teaching on learning strategies. The subjects were sophomore students majoring in Electrical Engineering in a national senior industrial vocational school located in southern Taiwan during the 2019 academic year. This study randomly selected two classes in the school. One class was the control group (*n* = 43) and the other was the experimental group (*n* = 42). The experimental group was instructed with the flipped teaching model, while the control group was under the traditional teaching method. The experimental group was divided into groups of three or four students, and the course lasted for 30 weeks (120 classes). This study collected relevant data before and after the teaching experiment, and open-ended questionnaires were used to collect qualitative data. 

This study employed the “Assessment Scale for Learning Strategies of Students in Vocational High Schools” [10], which has established national norms and proven to be effective. The definitions of the four criteria in the scale are as follows:

A. Learning motivations: motivations to achieve, and the degree of engagement when completing assignments;
B. Reading and tests: strategies and plans made for tests;
C. Self-testing: outstanding ability to review, ask questions, and complete exercises in class;
D. Problem-solving: noticeable ability to search for resources to solve problems.

After the flipped teaching, this study conducted a survey on the students. The questionnaire contains 24 items that evenly cover four sections of learning strategies for the electronics course, namely learning motivations, reading and tests, self-testing, and problem-solving. Each section contains 6 items.

### 3.1. Research Framework

**Figure 1:**
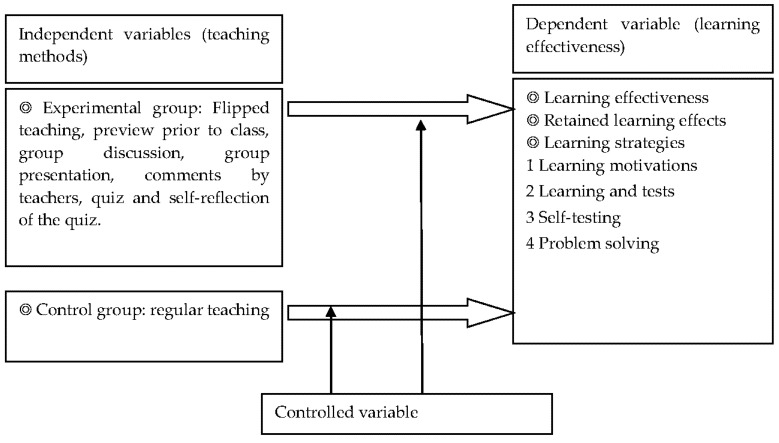
Study structure.

Based on the literature review, the research structure of this study, including controlled variables, independent variables, and dependent variables are shown in Figure 1.

The independent variables of this study are the teaching methods, i.e., the manipulated variables in the experiment to explore the learning effectiveness of subjects in all the Testing Center for Technological and vocational Education (here after as TCTE)-required courses regarding electronics. The experimental procedure of the flipped teaching method is illustrated in Figure 1.

There are three dependent variables: (1) learning outcomes: indicated by the post-test scores of the experimental group and control group after teaching; (2) retained learning effectiveness: indicated by the test scores of the experimental group and control group after a period of time following flipped teaching; (3) learning strategies: indicated by the students’ grades according to the Assessment Scale of Learning Strategies; a higher grade means stronger influences of flipped teaching on learning strategies. 

The controlled variables include student quality and faculty quality. In terms of student quality, the students in the experimental group and control group have equivalent academic performances. The admission grades of the two classes follow an “S” distribution, and their average grades in the Basic Electricity and Mathematics courses in the first year did not present obvious differences. In terms of faculty quality, while the teachers of the two classes both have over 15 years of teaching experience in the field of electronics, their teaching quality did not affect the experimental results. In addition, the two classes taught by the two teachers in the previous year did not show noticeable differences in the average grades of electronics in the National Model Exam.

### 3.2. Research Participants

The subjects were 85 sophomore students majoring in electrical engineering. All students had passed their basic entrance exam, and their admission grades in each class followed an “S” distribution. This study randomly selected one class as the control group (*n* = 43) and the other as the experimental group (*n* = 42). The teachers randomly assigned to the two classes have 15 to 25 years of teaching experience.

To ensure experimental accuracy, the experimental group and control group were not informed of the experiment. According to a survey of the students in the experimental class, they all had smartphones or computers at home to connect to the teaching platform. Therefore, it was expected that the experiment could be conducted smoothly.

### 3.3. Research Tools

This study adopted various teaching approaches, such as videos, tests, questionnaires, and semi-structured conversations, to collect, analyze, and arrange the data of a flipped electronics classroom. The study tools included learning materials for preview, the electronics study schedule, electronics testing, and the scale of learning strategies.

#### 3.3.1. Preview Materials

**Figure 2:**
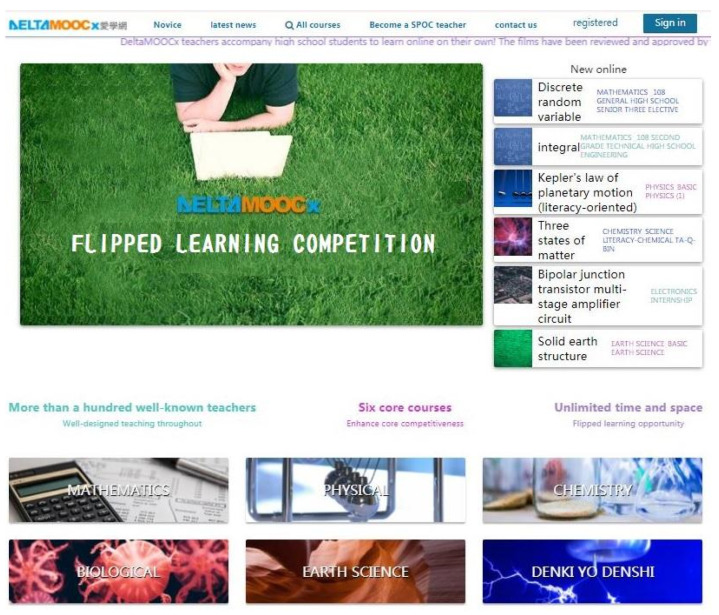
Screenshots of DeltaMOOC.

Based on the course objectives, this study selected preview materials from Delta MOOCx, (Delta Electronics Foundation, Taipei, Taiwan) which is a learning platform established by the Delta Electronics Foundation and Ministry of Education, as shown in Figure 2. MOOCX hosts many excellent teachers and professors of electronics from vocational schools and universities.

**Figure 3:**
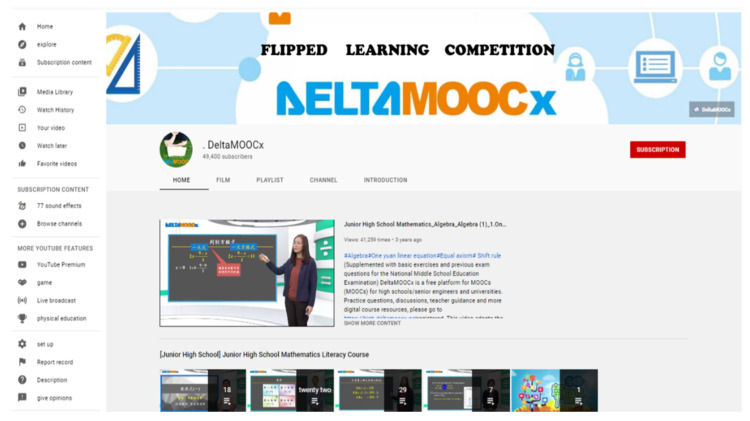
Screenshot of the homepage of the YouTube platform.

**Figure 4:**
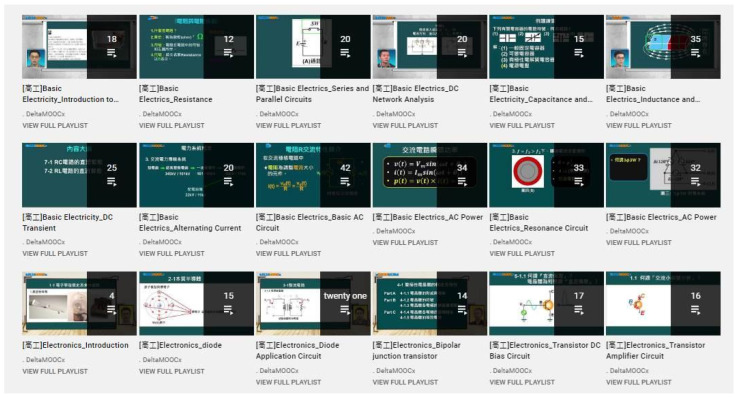
Learning contents of each electronics chapter.

As shown in Figure 3, the platform is accessible at https://www.youtube.com/ (accessed on 17 August 2020). With 188 classes, as illustrated in Figure 4, students could click and learn each chapter based on their needs.

#### 3.3.2. Learning Schedule

The design of the learning schedule in this study was based on the contents and learning objectives on the DeltaMOOCx platform, in order to easily check whether the students watched the videos, and whether they understood, memorized, contemplated, and wrote down the outline and key contents of the courses.

#### 3.3.3. Test of Electronics’ Knowledge

The test of this study covered the contents that had been taught to both experimental group and control group over a period of six weeks. The pre-test questionnaire contains 60 questions, which could be conducted again after the completion of the 6-week course. After examining the difficulty of the test, scholars and experts selected 40 out of 60 questions for the test.

During the last week of the second semester, meaning when both classes had finished the electronics course, the post-test was conducted, which was selected by experts from a past TCTE electronics exam.

#### 3.3.4. Assessment Scale of Student’s Learning Strategies

A. Selecting assessment scale:

This study adopted the learning strategy scale revised by Wu [10], which has been established as the national norm and features great reputation and effectiveness;

B. Effectiveness of the scale:

With 3321 valid questionnaires, the formal sample of this study was used to test the internal consistency and credibility of the sub-scale. The results show that the Cronbach’s α for the full assessment scale was 0.94, while the coefficients for the nine sub-scales ranged from 0.78 to 0.91, indicating good stability of the scale. The four sub-scales used in this study all had high correlations with the full scale: 0.71 for Learning Motivations; 0.75 for Reading and Tests; 0.82 for Self-Testing; and 0.78 for Problem Solving, embodying good internal correlations;

C. Assessment methods:

This study adopted a Likert-type assessment scale, where 1–5 points indicate the relevance between the test results and learning strategies. Through this method, this study investigates the usage situations of learning strategies in traditional and flipped classrooms.

During the teaching process, researchers constantly collected related data, including students’ interview contents, students’ feedback of the learning schedule, and teachers’ reflective journals, for the purpose of understanding the qualitative improvement of students’ learning. In addition to applying a questionnaire about the learning strategies, researchers also compared students’ grades in academic achievement tests. Understanding how flipped teaching improves learning effectiveness, and interactions between teachers and students would become a guide for researchers to ameliorate teaching methods.

## 4. Data Analysis and Discussion

This paper designed a flipped teaching course focusing on the subject of electronics in the vocational high school. Overall, students who participated in the course had significantly higher scores in the academic test than the control group. In terms of their reading and learning strategies, our results basically showed that the flipped teaching and the course design of electronics are in line with their learning strategies of motivation, self-testing, and problem solving. These findings were similar to other previous empirical studies flipped classroom courses [7,9]. A flipped classroom strategy is a student-centered active learning technique allowing students to focus on active learning and higher order thinking [11]. Similarly, the implication of flipped classroom strategy in teaching health care education also enables students to develop critical thinking, to rationally analyze medical evidence and utilize their knowledge of clinical care practically [24]. We discuss the results as follows.

### 4.1. Learning Effectiveness after Flipped Teaching

The experimental group and the control group both received traditional teaching on the subject of electronics the first six weeks of the experiment, and completed a pre-test at the end of the period. After 30 weeks, upon the completion of the course, both groups filled out the post-test.

The pre-test scores of the two groups were analyzed by the independent sample *t* test to determine the initial levels of the two groups in the subject of electronics. Then, the post-test scores were analyzed by the independent sample *t* test and compared with the pre-test scores, in order to understand the learning effectiveness of the two classes. Other differences between the two groups are illustrated as following Table 1.

#### 4.1.1. Analysis of the Pre-Test

The results show that the average scores of the two groups were very close, with the experimental group slightly higher than the control group. However, the statistics did not show whether there was significant difference between the initial scores of the two groups. Therefore, another round of independent sample *t* test was conducted, and the results are shown in Table 2.

As can be seen in Table 2, the F test was insignificant at *p* = 0.239 > 0.05. Assuming the variables were equal, *p* = 0.674 > 0.05, the initial scores of the two groups did not present noticeable differences. 

#### 4.1.2. Analysis of Post-test Scores

This study conducted the independent sample *t* test on the post-test scores of the two groups, in order to determine the influences of flipped teaching on students’ learning effectiveness.

Table 3 shows the means and standard deviations of the test scores of the two groups. The average score of experimental group was higher than that of the control group (49.14 > 29.67). Considering that statistics failed to prove significant differences in the test scores between the two groups, the independent sample *t* test was conducted, and the results are shown in Table 4.

Table 4 indicates that the F test was significant at *p* = 0.001 < 0.05. Assuming the variables were not equal, *p* = 0.000 < 0.05, the differences of the post-test scores between the two groups were not significant. In addition, as can be seen in Table 3, the average score of the experimental group was higher than that of the control group. Thus, the experimental group showed better learning effectiveness than the control group.

### 4.2. Retained Learning Effectiveness after Flipped Teaching 

The students in the experimental and control groups, who enrolled in the electronics courses for two semesters in the second year, took five national model exams in the third year. Moreover, they took a model exam every four months. This study used the scores of their first, third, and fifth national model exams for analysis to compare their retained learning effectiveness. The outcomes are as following Table 5.

A. Analysis of the scores of the first post-test

As shown in Table 5, the first post-test scores of the experimental group were 21.3 points higher than that of the control group. The results of the independent sample *t* test are shown in Table 6.

Table 6 shows that the F test was significant at *p* = 0.010 < 0.05. Assuming the variables were not equal, *p* = 0.000 < 0.05, it indicates significant differences between the first post-test performance of the two groups, meaning the experimental group outperformed the control group in terms of retained learning effectiveness.

B. Analysis of the scores of the second post-test

According to Table 7, the second post-test scores of the experimental group were 22.29 points higher than that of the control group. The results of the independent sample *t* test are also shown in Table 8.

Table 8 suggests that the F test was significant at *p* = 0.000 < 0.05. Assuming the variables were not equal, *p* = 0.000 < 0.05, it indicates significant difference between the second post-test scores of the two groups, meaning the experimental group outperformed the control group in terms of retained learning effectiveness.

C. Analysis of the scores of the third post-test

According to Table 9, the third post-test scores of the experimental group were 22.33 points higher than that of the control group. The results of the independent sample *t* test are shown in Table 10.

As shown in Table 11, the average score of the learning motivations in the post-test of the experimental group was higher than that of the pre-test (22.929 > 19.881), and the post-test scores were more concentrated (3.396 < 3.762). The mean of the reading and test scores of the experimental group were higher in the post-test (22.143 > 17.857), and more concentrated (3.530 < 4.076) than in the pre-test. The self-testing scores of the post-test were greater on average (21.309 > 17.667) and more concentrated (4.257 < 4.941) than that of the pre-test. In terms of problem solving, post-test scores were higher (21.619 > 19.214) on average and more concentrated (4.054 < 4.946) than the pre-test scores. However, as the results in Table 11 did not show significant difference, the pre-test and post-test results were analyzed by the dependent sample *t* test, as shown in Table 12.

Table 12 shows that the *p*-values for the experimental group’s learning strategy scale, as examined by dependent sample *t* test, were 0.000, 0.000, 0.000, 0.000, and 0.000, which are all less than 0.05. This indicates that the learning motivations, reading and test taking, self-testing, and problem solving of the experimental group have remarkable improvement after flipped teaching. In other words, applying flipped teaching in electronics courses is beneficial for students to strengthen their learning strategies.

D. Control group

According to Table 13, for the control group, the average score of learning motivations of the post-test was lower than that of the pre-test (19.302 < 20.070). The mean of reading and test scores of the control group were found to be slightly higher in the post-test than in the pre-test (18.674 > 18.372). The self-testing scores of the post-test of the control group were slightly greater on average than that of the pre-test (17.744 > 17.349). In terms of problem solving, the average of the post-test scores were lower than the pre-test scores (18.837 < 19.209). However, as Table 13 did not show significant difference, the results of the pre-test and post-test were analyzed by the dependent sample *t* testing, as shown in Table 14.

Table 14 shows that the *p*-values for the control group’s learning strategy scale, as examined by dependent sample *t* testing, are 0.180, 0.664, 0.525, and 0.552, which are all over 0.05, suggesting that the learning motivations, reading and test taking, self-testing, and problem solving abilities of the experimental group improved significantly after flipped teaching. This means traditional teaching in electronics courses did not improve students’ learning strategies.

Previous studies show [23] that during COVID-19 students generally were dissatisfied with online learning, particularly in the communication and Q&A modes. The combined model of online teaching with flipped learning may improve students’ learning, attention, and evaluation of courses [18,23,24]. Thus, this study aims to discuss whether flipped teaching could improve the learning effectiveness and learning strategies of the students, and even sustain and strengthen their learning strategies. To sum up, the findings of empirical studies confirm that flipped teaching enables us to engage students as the previous study’s demonstration [18], firstly to know new concepts related to electronics in advance and then to employ factual knowledge for understanding, references searching, and knowledge application during the class. In addition, the study also shows that students may employ metacognitive strategies after electronic class for self-learning through the change of study strategies. 

### 4.3. Qualitative Insights

This study has produced a set of open-ended interviewing questions on the course of flipped teaching and compiled the views and insights of the experimental group. In general, the researcher interviewed 42 students in the second semester and 40 of them had positive views on flipped teaching in the course of electronics. The students felt that pre-recording video and worksheet from the video would help them understand the content of electronics more quickly. However, there were also 2 students who had negative opinions, for example, they complained that pre-recording video lesson did waste their time for entertainment at home, and were tired of writing worksheets every week. Above finding is consistent with Ozdamli, Fezile, and Gulsum Asiksoy [25] that there are also certain students who decline to do homework, whether it is worksheet or a video lesson. Although previous studies have proven that students have merely 10 minutes of the introduction of a new topic before they lose interest [26,27,28]. Thus, we suggest that pre-recording video lesson should be limited as short minutes as possible.

In additional to maximizing the flexibility of flipped teaching characterized by teacher and student interactions and individualized learning as both pre-recorded video lesson and face to face lesson can be adjusted to suit the individual’s situation [26,27]. Most of the students mentioned that discussion in the class may increase the interaction among classmates, enhance the friendship of classmates, and allow everyone to express themselves courageously. Face-to-face time spent with both teachers and classmates is a beneficial for teachers as well since teachers do not have to repeat themselves in the class [26]. In the interview, we found that students with lower grades said that it was convenient to ask their classmates for advice on concepts they did not quite understand. Meanwhile, students with better grades said that teaching their classmates is also a way of learning, which enables them to review and clarify their concepts, and also to foster their expression skills. In a word, these discussions in the class may increase students’ problem-solving skills because most students do not want to lose to other groups. Above findings also echoed the statistics in Table 11 and Table 12, as mentioned previously in this paper.

With regarded to the development of study strategy, the teacher and the students communicate and discuss weekly progress plan thoroughly. In doing so, progress plan allows students to know the progress of the coming weeks. Each week there are pre-recording videos and worksheets, and each group discusses the progress and takes turns to present on the stage in the morning study three days per week. Students have to decide how to arrange their time after school and how to allocate their reading and leisure time. Teachers gave students more autonomy to prepare for formal exams. The teacher also asked the students to share their reading methods and plans in the classroom, and the majority of the students’ responses revealed that the group pressure made the students learn from each other, so there was a significant improvement in the development of study strategy.

In addition, during the flipped teaching process, the researcher made sure to teach the students to learn to use the headings and keywords of the text to identify the key points of lesson from each worksheet and textbook. At the end of the lesson, the researcher handed out a framework of practices to help students become more familiar with the topics. Before formal examination, the researcher discussed the key points of the lesson with the students. After formal examination, the students had established the process of reviewing the test questions. Finally, the results of the interviews showed that most of the students were positive about the process of presenting and solving problems in front of classmates, which helped them to understand more detail in terms of electronics. Our findings are also in line with previous [25,28,29] who suggested that through cooperative learning, group learning and sharing can leverage learning difficulties and increase group interaction.

## 5. Conclusions

This study explored the differences of flipped teaching and traditional teaching in terms of their influences on learning effectiveness and strategies of vocational students in electronics courses. The experimental results are as follows: firstly, flipped teaching has positive influence on the learning effectiveness of sophomore students in the electronics courses; secondly, flipped teaching positively influences sophomore students’ ability to retain the learning effectiveness in the electronics courses; thirdly, flipped teaching has positive influences on sophomore students’ learning strategies in electronics courses.

In addition, this study has shown that two full regular semesters of flipped classrooms enable vocational high school students to further improve their learning strategies. We echoed the previous study of Hew et al. [9] that the “new normal” of learning in pedagogical practices and learning performance in fully online flipped classrooms in the influence of COVID-19. In addition, to compare the short and few weeks curriculum, we found out that students‘ learning motivations, reading, and tests self-testing problem solving are improved in the fully regular online flipped teaching.

Overall, this paper suggested that flipped teaching could be the foundation of students’ self-regulation learning, which empowers students’ motivation and enables them to pursue a better academic performance. In other words, the flipped teaching model could increase possibilities of long term training or lifelong learning. However, the curriculum of this research is designed for a total of 2 full semesters. The limitation of this research is that it could benefit more on self-regulation learning but less on cognitive level of learning outcome. There were some limitations in this paper. First, the experimental design of compulsory education in Taiwan was not ideal because participants are not able to be randomly assigned. Second, the number of participants in this paper was low. Future use of this teaching model in a larger group is required to determine whether these results are generally consistent compared with qualitative data. Third, as teachers and students were participating in research, there might be a Pygmalion effect in which a student’s performance is affected by teacher’s expectations [30].

According to analysis and observations, the researchers propose the following suggestions for future studies: Firstly, More courses in the discipline of electronics could be included in the study, such as Basic Electricity, Digital Logic, and internship courses, to further explore the benefits of flipped teaching. Moreover, flipped teaching could be applied to many other subjects offered in vocational schools the future, such as electronics, information technology, industrial subjects, etc. The subjects of this study are sophomore students in a vocational high school, thus, future studies could include students from different grades or more classes to explore the effects of flipped teaching. In addition, future studies could apply other teaching methods in the experimental teaching, such as ShareStart teaching, or select different study regions, such as municipalities, county-administered cities, or rural areas, where the outcomes of flipped teaching may vary. Finally, schools of different scales, such as large, medium, and small schools, could be studied in order to compare the effects of flipped teaching. Finally, the learning strategies scale of this study only include four aspects, thus, future researchers can expand the aspects by strengthening teaching skills and strategies.

## Figures and Tables

**Table 1:** Pre-test descriptive statistics of the experimental group and the control group.

	Number	Mean	Standard Deviation	Standard Error of Mean
Experimental group	42	53.29	18.987	2.930
Control group	43	51.67	16.129	2.460

**Table 2:** Independent sample *t* test of the learning achievements of the pre-test of two groups.

Two Groups	Levene Test with Equal Variables		T Test with Equal Mean						
	F Test	Statistical Significance	*t*	Degree of Freedom	Significance (Two-Tailed Test)	Average Differences	Standard Errors	95% Confidence Interval	
								Lower Bound	Upper Bound
Assuming the variables were equal	1.409	0.239	0.422	83	0.674	1.611	3.818	−5.982	9.205

**Table 3:** Post-test descriptive statistics of the experimental group and the control group.

	Number	Mean	Standard Deviation	Standard Error of Mean
Experimental group	42	49.14	20.035	3.091
Control group	43	29.67	11.152	1.701

**Table 4:** Independent sample *t* test of the post-tests of the two groups.

Post-Test of Two Groups	Levene Test with Equal Variables		T Test with Equal Mean						
	F Test	Statistical Significance	*t*	Degree of Freedom	Significance (Two-Tailed Test)	Average Differences	Standard Errors	95% Confidence Interval	
								Lower Bound	Upper Bound
Assuming variables were not equal	12.591	0.001	5.518	63.861	0.000	19.468	3.528	12.419	26.517

**Table 5:** Descriptive statistics of the first post-test.

	Number	Mean	Standard Deviation	Standard Error of Mean
Experimental group	42	56.10	21.811	3.365
Control group	40	34.80	15.190	2.402

**Table 6:** Independent sample *t* test of the first post-test.

Pre-Test of Two Groups	Levene Test with Equal Variables		T Test with Equal Mean						
	F Test	Statistical Significance	*t*	Degree of Freedom	Significance (Two-Tailed Test)	Average Differences	Standard Errors	95% Confidence Interval	
								Lower Bound	Upper Bound
Assuming variables were not equal	7.062	0.010	5.151	73.385	0.000	21.295	4.135	13.056	29.535

**Table 7:** Descriptive statistics of the second post-test.

	Number	Mean	Standard Deviation	Standard Error of Mean
Experimental group	42	50.19	17.674	2.727
Control group	40	27.90	9.170	1.450

**Table 8:** Independent sample *t* test of the second post-test.

Pre-Test of Two Groups	Levene Test with Equal Variables		T Test with Equal Mean						
	F Test	Statistical Significance	*t*	Degree of Freedom	Significance (Two-Tailed Test)	Average Differences	Standard Errors	95% Confidence Interval	
								Lower Bound	Upper Bound
Assuming variables were not equal	19.079	0.000	7.217	62.229	0.000	22.290	3.089	16.117	28.464

**Table 9:** Descriptive statistics of the third post-test.

	Number	Mean	Standard Deviation	Standard Error of Mean
Experimental group	42	56.48	20.400	3.148
Control group	40	34.15	15.501	2.451

**Table 10:** Independent sample *t* test of the third post-test.

Pre-Test of Two Groups	Levene Test with Equal Variables		T Test with Equal Mean						
	F Test	Statistical Significance	*t*	Degree of Freedom	Significance (Two-Tailed Test)	Average Differences	Standard Errors	95% Confidence Interval	
								Lower Bound	Upper Bound
Assuming variables were not equal	3.703	0.058	5.559	80	0.000	22.326	4.016	14.334	30.318

**Table 11:** Descriptive statistics of learning strategy scale of experimental group.

	Semi-Aspects	Number	Mean	Standard Deviation	Standard Error of Mean
Experimental group	Pre-test of learning motivations	42	19.881	3.762	0.581
	Post-test of learning motivations	42	22.929	3.396	0.524
	Pre-test of reading and tests	42	17.857	4.076	0.629
	Post-test of reading and tests	42	22.143	3.530	0.545
	Pre-test of self-testing	42	17.667	4.941	0.762
	Post-test of self-testing	42	21.309	4.257	0.657
	Pre-test of problem solving	42	19.214	4.946	0.763
	Post-test of problem solving	42	21.619	4.054	0.626

**Table 12:** Dependent sample test of learning strategy scale of the experimental group.

	Semi-Aspects	Deviation Mean	Standard Deviation	*t*	Significance (Two-Tailed Test)
Experimental group	Learning motivations	3.047	3.774	−5.234	0.000
	Reading and tests	4.285	3.940	−7.049	0.000
	Self-testing	3.642	3.681	−6.413	0.000
	Problem solving	2.405	3.749	−4.158	0.000

**Table 13:** Descriptive statistics of learning strategy scale of control group.

	Semi-Aspects	Number	Mean	Standard Deviation	Standard Error of Mean
Control group	Pre-test of learning motivations	43	20.070	4.817	0.735
	Post-test of learning motivations	43	19.302	5.129	0.782
	Pre-test of reading and tests	43	18.372	4.836	0.737
	Post-test of reading and tests	43	18.674	4.927	0.751
	Pre-test of self-testing	43	17.349	5.126	0.781
	Post-test of self-testing	43	17.744	5.052	0.770
	Pre-test of problem solving	43	19.209	4.969	0.758
	Post-test of problem solving	43	18.837	5.141	0.784

**Table 14:** Dependent sample *t* test of learning strategy scale of the control group.

	Semi-Aspects	Deviation Mean	Standard Deviation	*t*	Significance (Two-Tailed Test)
Control group	Learning motivations	−0.767	3.689	1.364	0.180
	Reading and tests	0.302	4.257	−0.466	0.644
	Self-testing	0.395	4.048	−0.640	0.525
	Problem solving	−0.372	4.071	0.599	0.552

## Data Availability

The data presented in this study are available on request from the corresponding author. The data are not publicly available due to privacy and confidentiality for research participants.
